# Harnessing the disruption on medical trainee education due to COVID-19 in New South Wales, Australia

**DOI:** 10.12688/mep.19122.2

**Published:** 2022-11-22

**Authors:** Simone L. Van Es, Aaron J.H. Tan, Toni Vial, Jo Burnand, Claire M. Blizard

**Affiliations:** 1 NSW Health Education and Training Institute (HETI), Sydney, Australia; 2 School of Biomedical Sciences, Faculty of Medicine and Health, The University of NSW, Sydney, Australia

**Keywords:** medical education, digital learning, COVID-19, online engagement, medical trainee, doctor well-being

## Abstract

The coronavirus disease (COVID-19) pandemic has caused disruption and uncertainty for junior medical doctor training and education. This has compounded the existing stress experienced by this cohort. However, by choosing appropriate educational models, as well as using novel educational approaches and advancing our online technology capabilities, we may be able to provide acceptable and even, superior solutions for educational training moving forward, as well as promote trainee wellbeing during these uncertain times.

## Introduction

### Background: junior doctor training in New South Wales, Australia

In Australia, the Medical Portfolio in the New South Wales (NSW) Health Education and Training Institute (HETI) supports the education, training, and welfare of prevocational junior doctors in addition to many of the vocational training programs throughout the state of NSW. Using evidence-based approaches and evaluation findings, combined with educational design expertise, we develop contemporary and responsive educational support for these learner cohorts.

### Purpose of this paper

The purpose of this paper is to share some adaptations we have made to medical education for junior doctors, that has resulted from the disruption by the COVID-19 pandemic. Normalization Process Theory ^1^ tells us that adaptations made during disruption will continue to become embedded into practice over time. We discuss our adaptations to education and training over the last 3 years, experience and results arising from these changes, relevant support from the literature, and our plans to further embed these modifications moving forward.

### Medical training disruptions and stressors due to COVID-19

In Australia the psychological stress for junior doctors has been reported as up to 12 times higher than age-matched controls in other professions ^2^. COVID-19 has added to this existing psychological stress due to a number of reasons, including the increased demands and workplace redeployments, and the impact on their education and training ^2^. Further, the COVID-19 pandemic has resulted in disruptions to medical education and ‘training pipelines’ for junior medical doctors in Australia ^3^, similar to that experienced by trainees in other countries ^4^. Uncertainty about training progression and meeting training requirements during the pandemic has exacerbated this stress.

Self-determination theory explains that for a person to flourish they require three basic psychological needs: autonomy, competence and relatedness ^5^. Through isolation (physical, social and psychological), uncertainty and disruptions to training, as well as medical workforce redistributions, the impacts of COVID-19 challenge all of these basic needs in the junior doctor cohort. It can be argued that by delivering junior medical officer (JMO) education using emerging technology, aligning practice closely with popular educational theories, optimizing our skills as online educators, and constantly aligning our education delivery with trainee feedback and program evaluation findings, we may support these needs and advance the quality of our education and training programs.

Education needs to be a dynamic process that embraces innovation to adapt to changes in the user environment, as well as to constantly maintain engagement with our learners. This statement is reflected in the Von Restorff effect, where a stimulus or item that is different or novel will stand out more and will therefore be more likely to be remembered. Without innovative educational practice, the learning becomes repetitive with risk of saturation and potential for learner disengagement ^6^. Additionally, people learn in different ways and postgraduate university programs for clinicians report better participation rates and knowledge attainment when the students are offered a choice of how they can learn the same content ^7^.

### The partial shift to a virtual care health model during COVID-19

Part of the uncertainties and issues for junior doctor training during COVID-19 are the training gaps arising from the closing down of elective surgery, quarantine or illness of junior doctors and re-deployment ^3, 4^. In parallel with this COVID-19 has forced medical practice in Australia and other locations globally to shift to a partial virtual care model ^8, 9^, requiring modifications to how clinical interactions are carried out. The increased participation of trainees in telehealth consultations has been suggested by some groups as a potential solution to bolster the training experience needed for this cohort ^4^. However, this also adds to the pressure faced by trainees to upskill and refine their competencies in online communication and technology, particularly when past surveys have shown that lack of experience and training with telemedicine was one of the barriers to using telemedicine in practice ^9^. Consequently, offering education to trainees on how to optimally communicate and engage in an online environment is a valuable educational opportunity. Innovative and useful approaches on how to train and supervise in a virtual telemedicine setting have been recently described ^9^.

## Practical tips

By making learning personalized, engaging, connected, learner-centered and by prioritizing choice, we argue that our programs are responding to the training disruption and career uncertainty fueled by the COVID-19 pandemic. We discuss practical tips for adapting education and training, providing some examples from our own practice, where relevant, with support from the literature.

### Tip 1: Harness the evidence on online education to maximize the junior doctor learning, engagement and training experience

It has long been known that online learning can be efficient, effective, acceptable ^10, 11^ and sometimes even superior to traditional learning, particularly if interactive or engaging tools are used ^11^. Engagement can also be optimized in a virtual environment if the educational program is structured and facilitated in the right way ^12^.

Studies have shown both measurable and perceived benefits for adult learners using online learning ^10, 11, 13, 14^. This benefit is further augmented when the learning is active and delivered in a time efficient manner by an authoritative or trusted figure ^15^. There is an opportunity for a community of practice approach to learning in the online environment. Further benefits include equity of access, the perception of a personalized experience, and improved convenience of learning ^10^. The concept of “learning-on-the-go” can be facilitated by the native smartphone applications of some of the online communication and collaboration platforms in common use today. This would be a distinct advantage for junior medical trainees who may benefit from ‘just-in-time’ or ‘on-demand’ learning.

Timely feedback has been described as extremely helpful in the learning process, particularly if it is individualized, with explanations of associated issues or expected gold standard responses ^10^. If that feedback is also visually engaging, which can be easily facilitated with advancing digital technology, the learning experience is further enhanced ^10, 16, 17^.

Online delivery allows us to take advantage of engaging visual effects. The educational importance of this is described in dual coding theory, which asserts that we are more likely to remember images than words. A medical audience is also more likely to be engaged in their professional learning by images with judicious use of meaningful text compared to text alone ^6, 12, 18^. It captures attention better, and delivers complex concepts more effectively and efficiently. Information is twice as likely to be retained using this approach ^6^ and is also easily delivered with mobile technology. As a form of ‘just-in-time learning’, or ‘on-demand’ learning for busy junior doctors, infographics delivered on mobile devices are perceived as an effective way to learn by approximately 70% of surveyed junior doctors ^6^. Digital technology can facilitate improved visual design and aesthetics of learning, including well designed intuitive interfaces, all of which have a positive effect on learner engagement ^10, 12^.

To adapt to changing environment and learner, and use the evidence described above, we designed the “intern lecture series” followed by the “5-minute journal club” program

Initially at the start of COVID-19, weekly synchronous online 50-minute presentation sessions by discipline experts were organized as part of this program for junior doctors. Whilst the feedback from participants was highly positive, informally observed attendance diminished over time, down to 10 attendees for some sessions. Education needs to be dynamic and adapt to changes in user environment. Consequently, we redesigned our approach, converting the 50-minute intern lecture series for NSW junior doctors into a “5-minute journal club”. A journal article was summarized by a medical doctor each week, into approximately 5 points, accompanied by a visual graphic. The document was then shared via Microsoft SharePoint/Teams and delivered with a bit.ly shortened link allowing assessment of engagement using bit.ly analytics. This approach incorporates the concepts of cognitive load, just-in-time or on-demand learning, dynamic teaching, and dual coding theory. Uptake and engagement with the new approach was favorable. The number of clicks recorded to view the resource from week to week ranged between 32 to 204, with mean number of views at 131 clicks over the 3 month period.

Even though virtual journal clubs described in the literature have innovative aspects, they often pivot from the face-to-face to online environment using the traditional journal club community of practice structure ^19, 20^. In contrast, the ‘5-minute journal club’ represented a new educational development. Others have measured interest in their virtual journal club initiatives through innovative use of social media analytics ^19^. In contrast we chose a different approach measuring direct views of the resource through bit.ly analytics.

### Tip 2: Select the best-aligned virtual learning platform, technology and mode of delivery

Choice of a virtual learning platform to maximize trainee engagement and successful learning is important. Almarzooq and colleagues ^21^ started using Microsoft Teams (Microsoft Corporation, Redmond, Washington) for their cardiology trainees’ education during the early stages of the COVID-19 pandemic in the United States. They highlighted four key features for a successful virtual learning platform. These include integration, collaboration, education and communication features. Security of access to the communication platform is an important consideration when using clinical material in education sessions. This includes scenarios which require the direct involvement of consenting patients, such as during live-streamed ward rounds ^22^.

Another example of harnessing the evidence from the literature to select best-aligned technology and delivery is our ‘Surgical Sciences Intensive Course” (SSIC) pilot in 2021. This course supported junior doctors in their preparation for the Generic Surgical Science Examination (GSSE) for the Royal Australasian College of Surgery and was redesigned and delivered completely online in the COVID-19 environment in 2021. Based on positive feedback from participants, we continued the same approach in 2022.

The course caters for different adult learning styles, considered essential for effective adult education ^15, 23^. It combines recorded lectures from Subject Matter Experts (SMEs), pre- and post-course assessments, regular synchronous question and answer sessions with SMEs, and an intensive synchronous online anatomy weekend. The course is supported by a wide range of SMEs ensuring the engagement by the participants as literature has shown that engagement in education is maximized when delivered by trusted and authoritative figures ^15^.

Additionally, an observational educational approach, particularly when complemented with other forms of teaching, can be crucial for learning complex medical skills and knowledge ^23^. During this SSIC program we used this approach when delivering live streamed expert anatomy demonstrations, to geographically dispersed trainees, using high quality cameras with strategically placed overhead angles. This arrangement potentially provides the opportunity for trainees to experience clearer visual-spatial depiction of structures compared to observing in a more crowded group face-to-face setting. This approach can produce vivid imagery in the working memory, enhancing skill development ^23^.

In contrast to many other programs that were redesigned for online delivery ^24, 25^ the SSIC program assessed changes in knowledge or skill rather than purely user perception of benefit. Of the thirty two participants who sat the exit examination for this program, 20 (62.5%) showed an improvement in two or more subject areas of anatomy, pathology and physiology. Our findings are in-line with results of others who have found non inferiority of virtual delivery for teaching surgical skill subject matter ^26^. Additionally, 92% of our participants agreed that the new online pilot format of the course was an effective (23 of 25 respondents) way to learn.

Executive level support in our institution for development of this course was essential as initial costs were significant. However, as the course is now established future financial resources required will be minimal. The course structure is robust, in that it is evidence-based and supported by positive participant feedback, indicating sustainability through further challenges presented to the medical workforce by the COVID-19 pandemic.

### Tip 3: Use the technology for connecting emotionally and socially to maximize learning and development

Connecting emotionally and socially is an important way to support the psychological well-being of trainees. It is an essential part of the learning process, and a facilitator of professional identity development in doctors. Promoting positive emotional states can have a strong influence on cognitive processes, including attention, learning, memory and broader flexible problem solving ^27^. Surveys of Australian and New Zealand university educators ^14^ and both undergraduate ^13, 14^ and postgraduate ^28^ students early in the COVID-19 pandemic indicated many positive perceptions of a remote online learning experience. These included a perceived increased sense of safety, as well as perceived enhanced lines of communication with supervisors and teachers ^13^, with many feeling they received more rapid responses to their questions in online learning discussion boards in the fully online learning environment. However, there was also unfortunately a strong perceived lack of social connection with peers and supervisors, in the early COVID-19 pandemic online learning environment, resulting in feelings of isolation, which then translated into reduced motivation and poor time management ^13, 14^.

The risk for negative emotional effects associated with remote online learning can be mitigated through building strong connections within the learner group at the start of a program, ideally with facial visualisation, and an online learning community that will support trainees to keep them on track ^29^. The value of some form of facial visualisation, as well as social connection as part of the learning environment in postgraduate medical education, is suggested by the findings of Noguchi and Stanaway ^7^. When clinicians had a choice of participation method in a graded postgraduate course, although the online non-collaborative learning approach appeared most popular overall, there was higher uptake of the graded intense weekend face-to-face program, compared to a more extended delivery with online asynchronous collaborative aspects ^7^. Participation rates and grades were also better with the intense face-to-face delivery compared to online non-collaborative and online collaborative approaches ^7^. We have recently taken advantage of these findings from the literature, in our planning for a future Medical Leadership and Management course, where we have prioritized a foundational face-to-face session prior to the roll out of the rest of the program online.

Telehealth and virtual care are also increasingly part of the healthcare landscape, so supporting doctors with ways to connect socially and emotionally with their colleagues, mentors and patients in this new environment is important. This may also help strengthen professional identity development for junior doctors in these new online environments. 

Providing a learning group with a dedicated communal multifunctional virtual space may be important. Such a space might include a channel where the group meets for formal scheduled teaching sessions, as well as a separate but parallel channel, providing opportunity for trainee group chat, group study and collaboration on a 24/7 basis. By following this approach during the pandemic, supervisors of cardiology trainees in the United States perceived an increased quality and consistency in the education they provided, and by fostering a sense of learning community amongst their online trainees through use of Microsoft Teams, they perceived reduced trainee burnout and improved ‘wellness in a time when isolation has become a part of everyday life’ ^21^.

The Medical Portfolio at HETI runs The NSW JMO (Junior Medical Officer) Forum, composed post graduate year one (PGY1) and post graduate year two (PGY2) representatives from metropolitan, outer metropolitan, rural and remote hospitals. The purpose of the Forum is to provide a mechanism for representatives to collaborate and share innovations between hospital networks and discuss and action issues relevant to prevocational supervision, education and training, accreditation and welfare. During 2020 and 2021 the regular Forum meetings shifted to an online Zoom delivery. It continues in a hybrid fashion to support equity and inclusion for those working in outer metropolitan and rural and remote locations. In line with the evidence from the literature discussed, the junior doctors in this Forum are supported to thrive in the digital environment through presentations by subject matter experts on topics such as “successful online engagement” and “presenting your publications and posters online”, where the literature on innovations such as visual abstracts and infographics are also discussed.

### TIP 4: Rethink the Assessments

To some extent, we can learn from the reported experience of groups who conducted assessments for medical students during the early phases of the COVID-19 pandemic. Clinical assessments via objective structured clinical examinations (OSCE) and other telehealth assessments in the US ^30, 31^ and UK ^32^ respectively, were successfully delivered via Zoom™ (Zoom Video Communications Inc., San Jose, CA, USA) or Microsoft Teams (Microsoft Corporation, Redmond, Washington). Consistent and detailed communication between organizers, examiners and participants about the organization and technology were described as the keys to success.

In Australia there are over 21,000 vocational specialist training positions ^33^. Providing authentic assessments for these trainees during the COVID-19 pandemic was challenging and disruptive, particularly for large-scale barrier examinations. Positively, in Australia at least, this challenge and disruption has triggered opportunities to review and improve current systems. This includes re-thinking the reliance on high stakes barrier examinations for trainee progression, the associated risks of reliance on technology for these exams, and the security concerns and contingencies for technology failure during exam sittings ^33^. Suggested improvements emerging through responding to these challenges include introduction of assessments that are more flexible, as well as more aligned to trainees’ future practice and service delivery, including work place assessments, tele-supervision, online teaching and virtual assessment ^34^. The increased focus on virtual care during COVID-19 ^4, 8, 9, 34^ has also highlighted the importance of integrating education and assessment on digital and tele-health practice going forward ^34^. Our educational support in online communication and engagement skills for both trainees and educators is discussed in this paper.

### TIP 5: Train online educators

Using experienced and engaging clinical teachers, who are trained and skilled in online education and facilitation and have a strong online presence (such as using a conversational tone, using a photo for the educator’s online profile, being comfortable with using technology that will keep learners focused), leads to more engaged and motivated learners ^12, 22^. Further, these skills are readily transferable to the clinical context and will be helpful for clinicians (including trainees) in conducting the increasingly utilized telemedicine consultations.

Interactivity, intuitive tools, visually attractive learning resources, and personalization of the learning experience, have all been shown to improve engagement and facilitate understanding and knowledge retention ^10– 12^. Learners should be discouraged from ‘hiding’ in an online room, through requiring them to ‘do’ something at least every 5 minutes. Examples of such interactive activities might include commenting in the meeting chat or responding to polls.

Short introductory 2 minute videos on a theme or weekly topic in a course, where learning outcomes are discussed, enhances online teacher presence ^12^, creates structure and certainty for learners and may promote a sense of community for trainees. Encouraging trainees or learners to be partners in the learning and design process promotes empowerment and engagement ^12^.

Compulsory coursework is often used as a mechanism to ensure program engagement. Rather than providing incentive to engage, however, it may undermine learner autonomy and lead to reflex defiance and disengagement with the learning process ^5^. If elements of online course work are designed as compulsory components of the program, it may be important to provide adequate explanation as to why the component is compulsory, to support engagement and promote the sense of empowerment.

To support our clinical educators in online education and training, initiatives have included creating educational resources and guides in online engagement and teaching, interactive online presentations and webinars at HETI on how to maximize online engagement, as well as how to harness infographics and other visual learning content for this purpose. The resources are designed for both our medical specialist trainers and trainees. This approach has been underpinned by combining evidence from the literature with the practical education expertise within our organization.

### TIP 6: Manage learning curve expectations

Lack of both teacher and learner proficiency in effectively communicating in an online environment, as well as in using collaboration platforms and other digital technology, may represent an initial stumbling block. However, both speed and accuracy will increase with use and familiarity ^11^. Certainly, in the field of medical education, levels of acceptance and satisfaction with technology show steady increases with time ^11^. Findings are similar regarding learning curves for newly created virtual care clinics and newly implemented virtual supervision of medical vocational training and education ^8^. It is useful to point this out to trainees, so that the initial learning curves as well as subsequent improvements, will be an expectation if there is an extensive shift in delivery of the learning or if new digital approaches are being used.

### TIP 7: Incentivize online training or education programs

A primary challenge with any new technology or approach to learning is the group’s willingness to embrace the new technology or process ^21^. Whilst more than 50% of Australian postgraduate students prefer fully online courses, the retention rates for such courses are slightly less than hybrid courses and face-to-face courses ^28^. To manage this challenge, different incentives may be used to increase participation and completion rates for an online educational program ^29^. For tertiary education institution massive online open courses (MOOCs) where there was lack of credit associated with completing the course, dropout rates of 90% have been reported ^29^. When an incentive is introduced, such as credit towards graduation, then completion rates accelerated towards 70%. There are similar findings for postgraduate degree programs designed for Australian clinicians. Participation rates in their asynchronous online community learning components increased when incentive marks were given for written involvement in the course online learning forum ^7^.

### TIP 8: Manage fatigue, burn-out and cognitive load in online medical education programs

Time management is different for online learning when compared to traditional face-to-face methods. It takes more time to foster an effective remote learning environment, more time to deliver online content and more time to prepare or convert content for online delivery ^14^. This phenomenon of increased intensity associated with participating in remote online learning has been prominent enough since the early pandemic to coin the term of ‘Zoom fatigue’ ^14^. However, these issues can be mitigated by reducing online teaching for trainees into small digestible pieces, sometimes referred to colloquially as ‘chunks’, ‘packets’ or ‘bites’.

Educators might use existing curated quality collections of medical resources or images (existing radiology, pathology resources for example) ^14, 15, 35^. These strategies may reduce the intensity of the online learning load and increase both engagement and knowledge retention particularly when that small packet of knowledge is repeated at intervals ^35^. Traditional live hour-long lectures are becoming increasingly unpopular, regardless of whether they are delivered online or face-to-face. This is reflected by the dwindling attendance of students at live lectures when a recorded option will be available afterwards which can be watched at an accelerated speed and parts that need focus and reflection can be paused and re-listened to ^29^. Challa *et al.* ^23^ present evidence that the traditional didactic lecture needs to be replaced with a combination of innovative and modern learning systems which accommodate individual learning differences, and improve clinical reasoning and critical thinking. This may include combined approaches of eLearning, flipped classrooms, team-based learning and observational learning.

Further, 50- to 60-minute traditional teaching sessions are not compatible with the average human attention span and result in inferior engagement ^13, 14, 36^. This is compounded when one considers that learner focus is even shorter, when looking at a screen ^37^. Educational videos for online programs should be short productions of no more than 15 minutes ^37^. Simplicity of the learning design in an online learning experience is similarly important. This is reflected in the cognitive load theory ^38^, which describes how the average person can only process and retain three to four pieces of information at a time ^6, 39^. Consequently, an educational trend is emerging, where digestible educational ‘bites’ or ‘chunks’ of information of 6 to 15 minutes length are being delivered in online education and training programs ^29^.

To embrace these findings, in the last 12 months we have curated existing short 8 to 10 minute dynamic educational videos and delivered them once a week, to a cohort of junior doctors in NSW, who were part of a dedicated Microsoft Team. The approach was partly to manage cognitive load associated with learning, as well as to integrate effective learning into a fatigued workforce. The analysis of this project is in progress. Additionally, the “5-minute journal club” described in this paper aligns with the evidence from the literature discussed in this section.

## Conclusions

The COVID-19 pandemic has caused loss, disruption and uncertainty. It has brought us to a watershed moment in education and training for the medical profession. The most successful learning and teaching is dynamic, adapting to required change and producing something new. It can be argued that the adjusted educational approaches and technology that have emerged through the COVID-19 pandemic offer collaboration, improved educational delivery and engagement as well as sustainable support for the junior medical cohort. The COVID-19 pandemic has also positively triggered a re-thinking of training and assessment processes for junior doctors and specialist trainees in Australia.

Our article shares useful practical tips on eLearning formats that we have used to support for our junior doctor training including a “5-minute journal club”, surgical and anatomy skills development as well as training in communication skills for the virtual environment, with associated discussion from the relevant literature. Given the medical workforce has been disrupted in all countries and that there is similarity of training requirements for junior doctor cohorts globally, our experiences and findings may be helpful for other medical educator groups within Australia and as well as other countries.

We have shared some limited findings including anecdotal observations and brief analytics of participant use for these initiatives. It should be highlighted that these findings are sourced from internal program evaluation rather than formalized methodological research process and as such may represent a limitation of this practical tips paper. However, the lack of high-quality outcome data would be consistent with recently published literature ^24, 25^. Additionally, we emphasise that whilst some of our approaches are similar to those described in the literature, our experiences and recommendations shared within this paper may inform more formal study for the medical educational community. Lastly, in contrast to the majority of offerings globally, where educators ‘integrated technology to replace and amplify’ ^24^ we would like to highlight that we innovated and transformed many of our educational approaches in response to the increasing demand on our junior doctors and to support disrupted training as a result of COVID 19.

## Take home messages

- COVID-19 has had a marked impact on training, education and well-being for junior doctors in Australia as well as other countries.
- The disruption has resulted in positive shifts in educational practice, expectations and training frameworks for the junior medical workforce.
- Traditional education has been mostly abandoned to the emerging educational trend of using truncated, engaging and easily accessible ‘packets’ of medical practice-relevant educational content; using educators who are skilled in online engagement and who are perceived as authoritative figures by online learners is important.
- Subsequent work is emergent and evolutionary.
- We should continue to capitalize, at relatively low incremental cost, on the significant investment from the past 2 years in medical education and training adaptations
- Robust educational teamwork and leadership is essential in effecting successful innovative change for training and education of junior doctors.

## Data Availability

No formal data are associated with this article
