# Supplementary material for: Harnessing the disruption on medical trainee education due to COVID-19 in New South Wales, Australia
Source: MedEdPublish (2016). 2022 Nov 22;12:34. Originally published 2022 May 6. [Version 2] doi: 10.12688/mep.19122.2 (PMC10587661; doi:10.12688/mep.19122.2)
Supplement: Peer review history

# Reviewer report: Reviewer response for version 2

**DOI:** 10.21956/mep.20811.r34863

**Published:** 2023-10-09

**Authors:** Sana Saeed

**Affiliations:** 1 The Aga Khan University, Karachi, Sindh, Pakistan

Thank you for giving the opportunity to review this work. Following are the suggestions,

1. This is an experience of adapting the medical education for junior doctors specifically, hence some of the tips might not be generalizable. For example, TIP 7: Incentivize online training or education programs talks about adding incentives to improve participation, however, in medical education, assessment derives learning. It is suggested to change the title of the tip and make it more specific. 
2. Tip 6 is also confusing. Reading the tip header gives the impression that it is the time the doctor will take to learn the new concepts, however, the content of the tip is more focused on digital literacy. 
3. Tip 4: please provide the example from your context. What did you do and learn from? 
4. Tip 3 talks about using technology for connecting emotionally and socially, however the proposed solution is a face-to-face interaction. What is the role of technology in this context then?  
5. In Tip 2 , the authors claimed the change in the knowledge. Please elaborate on what assessment was used for this purpose. Also share the reliability and validity to strengthen the content presented in this tip. 

Is the topic of the opinion article discussed accurately in the context of the current literature?

Yes

Are arguments sufficiently supported by evidence from the published literature?

Partly

Are all factual statements correct and adequately supported by citations?

Yes

Are the conclusions drawn balanced and justified on the basis of the presented arguments?

Partly

Reviewer Expertise:

Curriculum development, teaching and learning, simulation-based learning, assessment and program evaluation

I confirm that I have read this submission and believe that I have an appropriate level of expertise to confirm that it is of an acceptable scientific standard, however I have significant reservations, as outlined above.

# Reviewer report: Reviewer response for version 2

**DOI:** 10.21956/mep.20811.r34296

**Published:** 2023-10-09

**Authors:** Yu-Che Chang

**Affiliations:** 1 Chang Gung Medical Education Research Centre; (CG-MERC), Chang Gung University, Taoyuan, Taoyuan City, Taiwan

1. This article has been submitted as opinion article but written as Practical tips in current revision. It is appropriate to categorized this article as a practical tips paper. I suggest to revise the article topic as “Eight tips for Harnessing the disruption on medical trainee education due to COVID-19 in New South Wales, Australia” or “Practical tips for Harnessing the disruption on medical trainee education due to COVID-19 in New South Wales, Australia” and also correct the article type as which is still in option article.
2. The article has written in very comprehensive way to share the experience about how to adapt to disruptions or uncertainties caused by COVID-19 pandemic on teaching and learning for junior doctor in NSW Australia. Most tips are written as reflection on curriculum evaluation and action point for readers based upon argument in the literature review. Even most outcomes are very descriptive and not solid in scientific level of evidence, this paper has been published in “Practical Tips” type and could still benefit clinical teachers and learners with future preparedness to respond to uncertainty and disruptions caused from COVID-19 impact.
3. The reference 5 aim to cite the definition of self-determination theory and I would suggest to cite this reference: Ryan, R. M., & Deci, E. L. (2017). *Self-determination theory: Basic psychological needs in motivation, development, and wellness.* The Guilford Press. https://doi.org/10.1521/978.14625/28806
4. The authors have addressed the adaptation to the challenge will benefit learners’ professional identity formation but lack of any reference in tip 5. Gagnon MP, Godin G, Gagné C, et al. An adaptation of the theory of interpersonal behaviour to the study of telemedicine adoption by physicians. Int J Med Inform. 2003;71(2-3):103-115. doi:10.1016/s1386-5056(03)00094-7 or Leslie, Katie F., et al. "The intersectionality of professional and personal identity formation in a virtual pre-health pathway program." *Currents in Pharmacy Teaching and Learning* (2023).

Is the topic of the opinion article discussed accurately in the context of the current literature?

Yes

Are arguments sufficiently supported by evidence from the published literature?

Yes

Are all factual statements correct and adequately supported by citations?

Yes

Are the conclusions drawn balanced and justified on the basis of the presented arguments?

Yes

Reviewer Expertise:

Professional identity, simulation-based medical education, competence-based medical education

I confirm that I have read this submission and believe that I have an appropriate level of expertise to confirm that it is of an acceptable scientific standard.

## References

[1] Self-Determination Theory: Basic Psychological Needs in Motivation, Development, and Wellness.2017; 10.1521/978.14625/28806 10.1521/978.14625/28806

[2] : An adaptation of the theory of interpersonal behaviour to the study of telemedicine adoption by physicians. *International Journal of Medical Informatics* .2003;71(2-3) : 10.1016/S1386-5056(03)00094-7 103-115 10.1016/S1386-5056(03)00094-7 14519403

[3] : The intersectionality of professional and personal identity formation in a virtual pre-health pathway program. *Curr Pharm Teach Learn* .2023;15(7) : 10.1016/j.cptl.2023.06.023 693-698 10.1016/j.cptl.2023.06.023 37394356

# Reviewer report: Reviewer response for version 2

**DOI:** 10.21956/mep.20811.r32790

**Published:** 2022-11-22

**Authors:** John Sandars

**Affiliations:** 1 Faculty of Health Social Care & Medicine, Edge Hill University, Ormskirk, UK

The authors have revised their submission and it now provides useful tips that will be of interest to a wider global audience.

Is the topic of the opinion article discussed accurately in the context of the current literature?

Partly

Are arguments sufficiently supported by evidence from the published literature?

Partly

Are all factual statements correct and adequately supported by citations?

Yes

Are the conclusions drawn balanced and justified on the basis of the presented arguments?

Partly

Reviewer Expertise:

Technology enhanced learning and educational interventions

I confirm that I have read this submission and believe that I have an appropriate level of expertise to confirm that it is of an acceptable scientific standard.

# Reviewer report: Reviewer response for version 1

**DOI:** 10.21956/mep.20480.r31969

**Published:** 2022-06-06

**Authors:** William Rainey Johnson

**Affiliations:** 1 Uniformed Services University of Health Sciences, Bethesda, MD, USA

Capturing the educational changes that have occurred during the COVID-19 pandemic is critical for establishing new best practices, especially in undergraduate and graduate medical education. I believe that this is an overarching goal of “Harnessing the disruption on medical trainee education due to COVID-19 in New South Wales, Australia”. The article offers insight into opportunities for educational transformation buried within an article that lacks identity. The article is written as an “opinion article”; however, I often found myself wondering whether this was meant as a review, practical tips piece, or a description of an advancement of instructional design. As a reader, I felt confused because of the lack of article identity and hope to point out some examples and opportunities for re-working the article to increase its impact on readers.

 The authors write that “the purpose of this paper is to reflect on the watershed moment in online medical education and training for junior doctors…due to the impacts of the coronavirus disease”. This purpose statement could more explicitly describe the authors’ intentions. I believe that their last sentence of the same paragraph more accurately captures the purpose, where they write that they “will discuss how best to shape and embed those adaptations, based on [their] experience and evidence from the literature”. I would suggest revising this sentence to make it the opening sentence of this paragraph and the explicit purpose of the paper.

 The authors proceed to provide some elements of review and some elements of practical tips without really committing to either. As an opinion piece, I was looking for the authors to have a strong thesis statement and convince me of the validity of their thesis through through literature and examples. I did not find this. Instead, I read discussions of educational theory, the published literature on changes in medical education during the COVID-19 pandemic, and changes in their educational practices. I suggest that the authors reorganize the article as a practical tips piece. While some literature has already been published on practical tips to online medical education (even some cited by the authors)(Reyna 2020 ^1^; Mooney et al. 2020 ^2^; Stojan et al. 2022 ^3^; Khamees et al. 2022 ^4^), the authors seem to have examples to strengthen suggestions that already exist in the literature, such as implementing “educational bites”, and other examples that add to the literature, such as their “5-minute journal club”.

 To reorganize into a practical tips piece, I would create headers with clear practical tips, such as “Train online educators with faculty development” (rather than “training online educators”). Beneath each practical tip, I would discuss the examples that the authors have already given from their practice and the supporting literature, much of which they have already cited.

 Ideally, the authors could provide stronger evidence to support the effectiveness of their changes, which may increase readers’ motivation to make changes within their institutions. For example, the authors write “the feedback from participants was highly positive” or “high evaluation response rates” in lieu of providing specific outcomes. If program evaluation data were collected, I encourage the authors to share it. If data were not collected, the authors should mention this as a limitation of educational initiatives. The lack of high-quality outcome data would be consistent with recently published literature (Stojan et al. 2022 ^3^; Khamees et al. 2022 ^4^).

 Finally, I would encourage the authors to consider a few additional references. There has been previous literature on e-learning formats for journal club (Lin and Sherbino 2015 ^5^; Belfi et al. 2021 ^6^) and surgical skills development(Nathan et al. 2022 ^7^), and anatomy teaching (Swinnerton et al. 2017 ^8^; Allsop et al. 2020 ^9^). Additionally, two relevant systematic reviews have been published (Stojan et al. 2022 ^3^; Khamees et al. 2022 ^4^).

Is the topic of the opinion article discussed accurately in the context of the current literature?

Yes

Are arguments sufficiently supported by evidence from the published literature?

Partly

Are all factual statements correct and adequately supported by citations?

Partly

Are the conclusions drawn balanced and justified on the basis of the presented arguments?

Partly

Reviewer Expertise:

Curriculum development, Self-monitoring, Graduate medical education

I confirm that I have read this submission and believe that I have an appropriate level of expertise to confirm that it is of an acceptable scientific standard, however I have significant reservations, as outlined above.

## References

[1] : Twelve Tips for COVID-19 friendly learning design in medical education. *MedEdPublish* .2020;9: 10.15694/mep.2020.000103.1 10.15694/mep.2020.000103.1 PMC1071260738090049

[2] : Rapid transition to online assessment: Practical steps and unanticipated advantages. *Medical Education* .2020;54(9) : 10.1111/medu.14225 857-858 10.1111/medu.14225 32403189

[3] : Online learning developments in undergraduate medical education in response to the COVID-19 pandemic: A BEME systematic review: BEME Guide No. 69. *Med Teach* .44(2) : 10.1080/0142159X.2021.1992373 109-129 10.1080/0142159X.2021.1992373 34709949

[4] : Remote learning developments in postgraduate medical education in response to the COVID-19 pandemic - A BEME systematic review: BEME Guide No. 71. *Med Teach* .2022; 10.1080/0142159X.2022.2040732 1-20 10.1080/0142159X.2022.2040732 35289242

[5] : Creating a Virtual Journal Club: A Community of Practice Using Multiple Social Media Strategies. *J Grad Med Educ* .2015;7(3) : 10.4300/JGME-D-15-00070.1 481-2 10.4300/JGME-D-15-00070.1 26457161PMC4597966

[6] : Virtual Journal Club Beyond the Pandemic: An Enduring and Fluid Educational Forum. *Current Problems in Diagnostic Radiology* .2021; 10.1067/j.cpradiol.2021.07.001 10.1067/j.cpradiol.2021.07.001 PMC842528834600795

[7] : Virtual Interactive Surgical Skills Classroom: A Parallel-group, Non-inferiority, Adjudicator-blinded, Randomised Controlled Trial (VIRTUAL). *J Surg Educ* .79(3) : 10.1016/j.jsurg.2021.11.004 791-801 10.1016/j.jsurg.2021.11.004 34857499

[8] : The integration of an anatomy massive open online course (MOOC) into a medical anatomy curriculum. *Anat Sci Educ* .2017;10(1) : 10.1002/ase.1625 53-67 10.1002/ase.1625 27315160

[9] : Using videoconferencing to deliver anatomy teaching to medical students on clinical placements. *Translational Research in Anatomy* .2020;19: 10.1016/j.tria.2019.100059 10.1016/j.tria.2019.100059

# Response

**Published:** 2022-11-11

**Authors:** Simone L. Van Es

**Affiliations:** The University of New South Wales, Australia

**Response to Dr Johnson** The authors are grateful for the insightful, detailed and constructive feedback from this reviewer. We have extracted some of the reviewer’s specific suggestions and responded to each of these suggestions.

- *” This purpose statement could more explicitly describe the authors’ intentions. I believe that their last sentence of the same paragraph more accurately captures the purpose, where they write that they “will discuss how best to shape and embed those adaptations, based on [their] experience and evidence from the literature”.*

The reviewer has made a good point. In response, we have created a specific paragraph early in the introduction in line with the terminology suggested. We feel this has improved the clarity of the introduction, thank you.  

- *“I suggest that the authors reorganize the article as a practical tips piece”.*

We found the reviewer’s comments on reorganising the article into a ‘practical tips’ format very useful. Whilst this represented a moderate change in the architecture of the paper, shifting to a “practical tips” approach presented the material more coherently, allowed us to relate our adaptations in practice to the existing literature and thus, as the reviewer suggested, allowed us to more clearly strengthen the material existing in the literature. The revised format of the paper aligns well to the criteria for a practical tips article. Thank you for that direction, it was a good idea.  

- *“To reorganize into a practical tips piece, I would create headers with clear practical tips, such as “Train online educators with faculty development” (rather than “training online educators”). Beneath each practical tip, I would discuss the examples that the authors have already given from their practice and the supporting literature, much of which they have already cited”.*

In line with the reviewer’s recommendations, we have created clear practical tips headers using the imperative from a grammatical perspective, as suggested, for example, “Tip 1: Harness the evidence on online education”, “Tip 3:  Use the technology for connecting emotionally and socially”, or “Tip 6: Manage learning curve expectations”.  Again this was a useful suggestion as it provides more clarity and direction to the paper. Under each heading, we give examples of our practice where relevant and support and link this to the literature, as was suggested.  

- *“If program evaluation data were collected, I encourage the authors to share it. If data were not collected, the authors should mention this as a limitation of educational initiatives”.*

In line with the reviewer’s recommendations, we have shared some limited findings and results including anecdotal observations and brief analytics of participant use for some of our educational initiatives. We however also highlighted in one of the final paragraphs that these findings and results are sourced from internal program evaluation rather than formalized methodological research process and as such may represent a limitation of this practical tips paper. As the reviewer pointed out this limitation is consistent with recently published literature.

- “ *Finally, I would encourage the authors to consider a few additional references* *”*

The authors would like to thank the reviewer for providing a range of useful recent references (and insightful relevant comments) that have strengthened this paper.

# Reviewer report: Reviewer response for version 1

**DOI:** 10.21956/mep.20480.r31968

**Published:** 2022-05-06

**Authors:** John Sandars

**Affiliations:** 1 Faculty of Health Social Care & Medicine, Edge Hill University, Ormskirk, UK

The authors present a description of their experience of the design and implementation of new approaches to educational training that they offered to junior doctors in Australia in response to the demands of the COVID-19 pandemic. There appears to be little evaluation of this approach, althought the authors state "The purpose of this paper is to reflect on the watershed moment in online medical education and training".

 The design of the new approaches are justified by the authors by making links to appropriate educational theories, 'best practices' and published studies. This information is useful to other educators but would be more useful if the extent to which these approaches are similar or different to other responses made during the COVID-19 pandemic could be clearly discussed. This information is required by other educators for making decisions about applying the presented approaches in their own context.

 The article is mainly descriptive but readers would be interested in more in-depth evaluation data on usefulness and ease of use by both educators and learners. This information is required by other educators for making decisions about applying the presented approaches in other contexts.

 Overall, this reflective and predominantly descriptive article provides a snapshot within one specific context but is limited in potential applicability for medical educators in other contexts.

Is the topic of the opinion article discussed accurately in the context of the current literature?

Partly

Are arguments sufficiently supported by evidence from the published literature?

Partly

Are all factual statements correct and adequately supported by citations?

Yes

Are the conclusions drawn balanced and justified on the basis of the presented arguments?

Partly

Reviewer Expertise:

Technology enhanced learning and educational interventions

I confirm that I have read this submission and believe that I have an appropriate level of expertise to confirm that it is of an acceptable scientific standard, however I have significant reservations, as outlined above.

# Response

**Published:** 2022-11-11

**Authors:** Simone L. Van Es

**Affiliations:** The University of New South Wales, Australia

**Response to Dr Sandars**  

 The authors are grateful for the thoughtful and constructive feedback from this reviewer. We have extracted some of the reviewer’s specific suggestions or comments  and responded to each of these suggestions or comments.  1. “ *This information is useful to other educators but would be more useful if the extent to which these approaches are similar or different to other responses made during the COVID-19 pandemic could be clearly discussed.* *”* The reviewer makes a good point. In this revised version of the paper, when we mention our practical educational innovations or adaptations, we have aimed to discuss whether this is line with other groups’ findings and thus supportive of the literature or whether our approaches are in contrast to approaches in the literature, and thus represent something more novel. For example, under “Tip 1”, we refer to other virtual journal clubs described recently in the literature. Under “Tip 2”, we discuss our Surgical Skills Intensive Course, and discuss how the evaluation of this course differs from  the majority of other  programs described  in the literature that had also been redesigned for online delivery. Additionally in the conclusion we discuss some of the limitations of our paper and how these limitations are consistent with recently published data. 2. “readers would be interested in more in-depth evaluation data” The reviewer makes a very valid point, thank you. In line with the reviewer’s recommendations, we have shared some limited findings and results including anecdotal observations and brief analytics of participant use for some of our educational initiatives. We however also highlighted in one of the final paragraphs that these findings and results are sourced from internal program evaluation rather than formalized methodological research process and as such may represent a limitation of this practical tips paper.
